# Effects of nutritional supplementation on glucose metabolism and insulin function among people with HIV initiating ART

**DOI:** 10.1186/s40795-021-00462-y

**Published:** 2021-10-18

**Authors:** Hiwot Amare, Mette F. Olsen, Henrik Friis, Pernille Kæstel, Åse B. Andersen, Alemseged Abdissa, Daniel Yilma, Tsinuel Girma, Daniel Faurholt-Jepsen

**Affiliations:** 1 Department of Internal Medicine, Jimma University, Jimma, Ethiopia; 2 JUCAN Research Centre, Jimma University, Jimma, Ethiopia; 3 Department of Nutrition, Exercise and Sports, University of Copenhagen, Copenhagen, Denmark; 4 Department of Infectious Diseases, Heart Centre, Copenhagen University Hospital Rigshospitalet, Copenhagen, Denmark; 5 Department of Laboratory Sciences, Jimma University, Jimma, Ethiopia; 6 Department of Pediatrics and Child Health, Jimma University, Jimma, Ethiopia

**Keywords:** Lipid-based nutritional supplements, Soy, Whey, Glucose, Insulin, HIV, Ethiopia

## Abstract

**Background:**

Without high-quality nutritional support, there is a risk that people infected with human immunodeficiency virus (HIV) will replace lost muscle mass with fat mass when initiating antiretroviral therapy (ART). We have shown that lipid-based nutrient supplements (LNS) with whey or soy considerably increases lean mass among Ethiopian people with HIV starting ART. Here, we aim to assess the effects of LNS on insulin function and glucose metabolism.

**Methods:**

This is a secondary analysis of a randomized trial testing the effect of three-month supplementation with LNS containing whey (LNS/whey) or soy (LNS/soy) among people with HIV. LNS/whey and LNS/soy groups were combined and then were compared against the non-supplemented group. The outcomes were change in fasting plasma-glucose (FPG), and 30-min glucose and 120-min glucose after oral glucose tolerance test. We further assessed effect on glycated hemoglobin (HbA1c), fasting insulin, homeostatic model assessment index for beta-cell function (HOMA-B) and insulin resistance (HOMA-IR).

**Results:**

Of the 318 patients enrolled, 268 (84.3%) had available FPG and HbA1c and included. After 3 months of ART, HbA1c tended to be 2 mmol/mol higher in the LNS supplemented group, most pronounced among those receiving whey as the protein source. LNS led to higher 30-min glucose (0.5 mmol/L, 95% CI 0.2, 0.8) and 120-min glucose (0.4 mmol/L, 95% CI 0.03, 0.8) and a > 50% increase in fasting insulin, HOMA-B and HOMA-IR compared to the non-supplemented.

**Conclusion:**

Among Ethiopian people with HIV initiating ART, short-term LNS intake increased glucose and insulin levels, and tended to increase HbA1c, potentially leading to more insulin resistance. Higher intake of carbohydrates with LNS could influence glycemic status. Whether these metabolic changes in early HIV treatment are beneficial or increase long-term risk of metabolic disorders needs to be explored.

**Supplementary Information:**

The online version contains supplementary material available at 10.1186/s40795-021-00462-y.

## Introduction

The human immunodeficiency virus (HIV) pandemic has widely affected Africa in the past 30 years, however, the care of HIV in Sub Saharan Africa has improved with the wide provision of antiretroviral therapy (ART) [1] and integration of HIV programs in the health care systems [2].

The prevalence of metabolic syndrome and non-communicable diseases (NCDs) is increasing in developing countries [3–6], and not least among adults with HIV [6]. Lipodystrophy, increasing life expectancy and some of the antiretroviral drugs, may put people with HIV at increased risk [7]. Furthermore, childhood under-nutrition leads to increased fat mass at the expense of lean mass and may therefore increase the risk of NCDs [8]. Most studies on diabetes and HIV are performed in high-income countries, where risk factors such as smoking and obesity are common in addition to the HIV-related risk factors [9]. In Ethiopia, the prevalence of obesity and smoking is low and malnutrition is high among the general population [10], but there is little knowledge of the nutritional impact on risk of diabetes among people with HIV.

Nutritional supplementation has been found useful in delaying progression of HIV disease [11], improving immune recovery [12], gain of lean mass and grip strength [13] and quality of life [14]. Consequently, nutritional supplementation has been integrated into numerous HIV care guidelines [15]. With increasing use of nutritional supplements resulting in weight gain, it is likely that altered body composition will affect glucose metabolism and insulin function and hence risk of diabetes among people with HIV on ART.

We conducted a nutritional supplementation trial (the ARTFOOD study) to investigate the effects of lipid-based nutrient supplements (LNS) among adults with HIV in Ethiopia. As previously reported, LNS supplementation for 3 months together with ART initiation improved weight, lean body mass, muscle strength, and the findings also suggested a positive effect on immune recovery [13]. As treatment guidelines have changed with early ART initiation, it is no longer possible to conduct new trials in late presenting patients with advanced HIV making our existing data unique for assessing effects in relatively immunocompromised individuals with HIV. Thus, based on the ARTFOOD trial data, this secondary analysis of a randomized trial investigates the effect of LNS on glucose metabolism and insulin function as indicators of diabetes risk in people with HIV with compromised immunity.

## Methods and settings

### Study setting and participants

The ARTFOOD study was conducted from 2010 to 2013 and included a total of 318 individuals with HIV. Participants were recruited from Jimma University Specialized Hospital, and Agaro and Jimma Health Centers, Ethiopia. Adults with HIV eligible for ART initiation, with body mass index (BMI) ≥17 kg/m^2^ were included [13]. Participants living outside 50 km radius of the recruitment centers or taking other nutritional supplements, pregnant or lactating women and people with known diabetes were excluded from the study [13]. If both glycated hemoglobin (HbA1c) and fasting plasma glucose (FPG) at baseline were missing, the participant was excluded from the present report. The HIV staging was based on the World Health Organization (WHO) clinical staging and the ART initiation was based on the national guideline at the time: CD_4_ count < 200 cells/μl regardless of clinical features, WHO clinical stage-III if CD_4_ count < 350 cells/μl and stage-IV irrespective of the CD_4_ count [16]. The national first-line ART regimens included zidovudine, lamivudine, tenofovir, efavirenz and nevirapine in different combinations and based on availability [16]. Isoniazid prophylaxis for tuberculosis and co-trimoxazole prophylaxis for *Pneumocystis jiroveci* pneumonia prophylaxis was provided. Full tuberculosis treatment, if relevant, was offered according to national guidelines [16].

### Demographic and clinical data

Demographic data were collected using a structured questionnaire by trained nurses. The questionnaire was translated into Amharic and Afaan Oromo. HIV-related clinical data concerning HIV WHO staging and opportunistic infections were collected using medical history and clinical examination.

### Study design and randomization

**Fig. 1:**
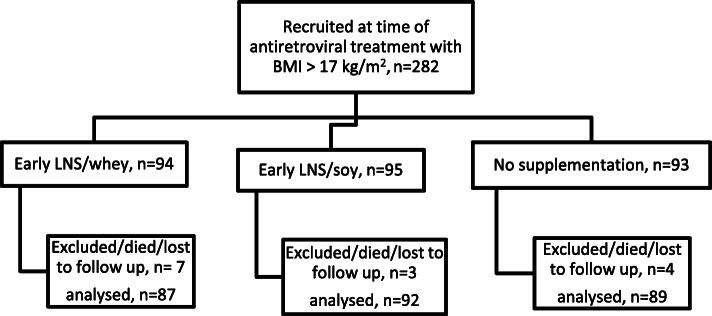
Flow diagram of randomization to LNS with either whey or soy supplementation or no supplementation

The ARTFOOD trial was a single-blinded randomized controlled trial [13] with participants randomized 2:1 to receive LNS or no nutritional supplementation for the first 3 months of ART. Those receiving LNS were randomized 1:1 to receive LNS with either whey (LNS/whey) or soy (LNS/soy) as protein source [13]. The design of the study has been reported in detail elsewhere [13]. In the current secondary analysis of the trial, LNS/whey and LNS/soy groups were combined and were considered as the supplemented group. Then, the supplemented group were compared against the non-supplemented group (Fig. 1).

### Nutritional supplementation

A daily supplement of 200 g (4600KJ) LNS was given [13]. It was produced by Nutriset® (Malaunay, France). The energy distribution of the supplements included 60% fat (palm and rapeseed oil plus peanuts), 24% carbohydrates (saccharose and maltodextrin), 16% protein; with either whey or soy as the protein source. During the study, supplements were provided on monthly basis. Adherence was assessed from returned empty sachets, self-reported consumption, and a qualitative sub-study. Poor adherence was defined as adherence to LNS < 75% [13]. Other details of the supplementation have been presented previously [13, 17].

### Outcomes

The outcomes were effect of LNS on change in FPG, HbA1c, fasting insulin as well as 30-min glucose, 120-min glucose, measured after a standard oral glucose tolerance test (OGTT). We further assessed the effect of LNS on changes in homeostatic model assessment index for beta-cell function (HOMA-B) and insulin resistance (HOMA-IR). All outcomes were assessed at baseline and after 3 months ART.

### Oral glucose tolerance test and laboratory data

A standard 75 g OGTT was performed using 82.5 g glucose monohydrate (Fagron, Netherlands) diluted in 250 ml drinking water and taken by the participant within 5 min. The participant did not take food or other drinks during the two test hours. Glucose was measured fasting, 30 and 120 min after administration of the glucose solution. All Glucose measurements were determined from whole blood and converted to plasma equivalents using the Hemocue Glucose 201 RT system (Hemocue®, Ängelholm, Sweden). Diabetes was defined according to WHO guidelines [18]. Fasting insulin (μIU/mL) was measured using a commercial ELISA kit (DAKO code K6219, DAKO, Glostrup, Denmark). HOMA-B and HOMA-IR were calculated by using the FPG and fasting insulin using standard formulae [19]. HbA1c (mmol /mol) was measured in fresh (within 30 min) EDTA stabilized blood on a Quo-test A1C analyzer (Quotient Diagnostics, Surrey, UK). Based on HbA1c, pre-diabetes and diabetes were defined as HbA1c 39 to 47 mol/mol and ≥ 48 mmol/mol, respectively [20]. C-reactive protein (CRP) was measured in serum using a latex enhanced immunoturbidimetric assay (HORIBA ABX A11A01611) for Pentra 400 (HORIBA ABC, Montpellier, France).

### Data analyses

Data were double entered and validated using EpiData (EpiData Association, Odense, Denmark). Data analysis was performed using STATA version 12.0 (StataCorp LP, College Station, TX). Intention to treat principle was used based on available cases. Comparisons between supplemented and non-supplemented groups were performed using one-way ANOVA. Linear regression models adjusted for age, sex, and baseline values of outcome variables were used to assess the effect of supplementation on glucose and insulin markers after 3 months treatment. Log10 transformations were used for fasting insulin, HOMA-B and HOMA-IR prior to regression to achieve a normal distribution. Back-transformed values are provided and the difference between groups expressed in the beta coefficient (10^B^) should be interpreted as a ratio relative to the non-supplemented group. Those with missing data in any of the outcome variables were removed from the final data analysis. All tests were two-sided and *p*-values < 0.05 were considered significant.

## Results

Of the 318 patients enrolled, 268 (84.3%) had available FPG and HbA1c and included in this report. The mean (±SD) age was 33 (±9) years, mean BMI was 19.8(±2.3) kg/m^2^ and 180 (67.2%) were women. The median (IQR) CRP was 1.8 (0.6–6.4) mg/L. There was no difference in the baseline characteristics between those randomized to LNS supplementation and non-supplementation (Table 1 and Supplemental table 1). The median adherence to the supplements was 86% (IQR 71–95%).

**Table 1:** Baseline characteristics of 268 HIV patients at time of initiation of antiretroviral therapy randomized to lipid-based nutrient supplements or no supplements

Characteristics	Non-supplemented (***n*** = 89)^a^	Supplemented (***n*** = 179)^a^
Age (years)	32 ± 9	33 ± 9
Women, n (%)	63 (70.8)	117 (65.4)
Educational status		
No schooling, n (%)	27 (30.3)	40 (22.4)
Primary education, n (%)	44 (49.4)	103 (57.5)
Secondary and higher education, n (%)	18 (20.2)	36 (20.1)
Body mass index (kg/m^2^)	19.8 ± 2.1	19.9 ± 2.3
Weight (kg)	51.4 ± 7.3	54.2 ± 7.4
Waist circumference (cm)	71.9 ± 6.7	73.0 ± 6.5
C-reactive protein (mg/L)^b^	2.1 (0.6–7.5)	1.6 (0.5–5.5)
**HIV-related characteristics**		
WHO stage		
Stage I, n (%)	25 (28.4)	61 (34.1)
Stage II, n (%)	26 (29.6)	58 (32.4)
Stage III, n (%)	29 (33.0)	44 (24.6)
Stage IV, n (%)	8 (9.1)	16 (8.9)
HIV viral load (log (1+ copies/mL))	4.7 ± 0.8	4.8 ± 0.8
CD4 count (cells/μl)	191 ± 107	185 ± 101
CD8 count (cells/μl)	878 ± 416	871 ± 426

*Abbreviations*: *HIV* Human immunodeficiency virus, *LNS* Lipid-based nutrient supplements, *WHO* World Health Organization

^a^Data are means (±Standard deviation) or number (%)

^b^Data presented with median with interquartile range

At baseline, prior to ART initiation, there was no difference in glucose and insulin markers between LNS supplemented and non-supplemented participants (Table 2). Three months after initiation of ART, levels of 30-min and 120-min glucose, but not FPG, were higher in the supplemented group compared to the non-supplemented (Table 3). Supplementation tended to lead to higher HbA1c (2 mmol/mol, 95% CI-0.1, 4.1, *P* = 0.067). Compared to non-supplemented, there was an increase of all insulin markers (fasting insulin, HOMA-B and HOMA-IR) in the supplemented group. Insulin markers were higher in the supplemented group by 54–64% compared to non-supplemented groups. There was no difference between the unadjusted and the adjusted models (data not shown).

**Table 2:** Unadjusted means of glucose and insulin markers among 268 HIV patients at baseline before initiation of antiretroviral therapy grouped by allocation to lipid-based nutrient supplements or no supplementation

	Non-supplemented (***n*** = 89)	Supplemented (***n*** = 179)
	Means (95% CI)	Means (95% CI)
Glycated hemoglobin, mmol/mol	36.1 (34.2; 37.9)	35.9 (34.6; 37.2)
Plasma glucose, mmol/l		
Fasting	5.6 (5.5; 5.8)	5.8 (5.7; 5.9)
30 min^a^	7.4 (7.1; 7.6)	7.9 (7.7; 8.1)
2 h^a^	6.3 (6.0; 6.6)	6.7 (6.5; 6.9)
Fasting plasma-insulin, μIU/ml^b^	3.4 (2.8; 4.2)	3.1 (2.6; 3.7)
HOMA-B^b^	35.0 (27.7; 44.3)	32.3 (26.8; 39.0)
HOMA-IR^b^	0.8 (0.7; 1.0)	0.8 (0.6; 0.9)

Beta estimates are presented after a linear regression with 95% confidence interval

Data available: HbA1c: 227 at baseline and 192 at 3 months. Fasting: 264 at baseline and 249 at 3 months. 30 m-PG: 264 at baseline and 248 at 3 months. 2 h-PG: 265 at baseline and 247 at 3 months. Plasma fasting insulin: 265 at baseline and 246 at 3 months

HOMA-B: 262 at baseline and 246 at 3 months. HOMA-IR: 263 at baseline and 246 at 3 months

*Abbreviations*: *CI* Confidence interval, *HIV* Human immunodeficiency virus, *HOMA-B* Homeostatic model assessment of beta cell function and *HOMA-IR* Insulin resistance

^a^Measured after oral glucose tolerance test

^b^Geometric means are used for skewed distributions

**Table 3:** Effect of lipid-based nutrient supplements on change in the glucose and insulin markers among 268 HIV patients after 3 months of antiretroviral therapy initiation

	Non-supplemented (reference, ***n*** = 89)	Supplemented (***n*** = 179)	Difference	
	**Adjusted means (95% CI)**	**Adjusted means (95% CI)**	**B (95% CI)**	***P*** **value**
Glycated hemoglobin, mmol/mol	34.0 (32.2; 35.7)	35.9 (34.8; 37.1)	2.0 (−0.1; 4.1)	0.067
Plasma-glucose, mmol/l				
Fasting	5.7 (5.5; 5.8)	5.8 (5.7; 5.9)	0.1 (−0.04; 0.3)	0.145
30 min	7.4 (7.2; 7.7)	7.9 (7.7; 8.1)	0.5 (0.2; 0.8)	0.003
2 h	6.3 (6.0; 6.6)	6.7 (6.5; 6.9)	0.4 (0.03; 0.8)	0.035
	**Adjusted geometric means (95% CI)**	**Adjusted geometric means (95% CI)**	**10**^**B**^ **(95% CI)**	***P*** **value**
Fasting plasma-insulin, μIU/ml^a^	3.3 (2.7; 4.1)	5.2 (4.4; 6.4)	1.57 (1.20;2.05)	0.001
HOMA-B^a^	31.2 (25.0; 39.0)	48.2 (41.1; 56.4)	1.54 (1.17; 2.03)	0.002
HOMA-IR^a^	0.80 (0.64; 1.00)	1.31 (1.12; 1.54)	1.64 (1.25;2.15)	< 0.001

Beta estimates are presented after a linear regression with 95% confidence interval adjusted for age, sex, educational status and baseline status of outcome variables

Data available: HbA1c: 227 at baseline and 192 at 3 months. Fasting: 264 at baseline and 249 at 3 months. 30 m-PG: 264 at baseline and 248 at 3 months. 2 h-PG: 265 at baseline and 247 at 3 months. Plasma fasting insulin: 265 at baseline and 246 at 3 months

HOMA-B: 262 at baseline and 246 at 3 months. HOMA-IR: 263 at baseline and 246 at 3 months

*Abbreviations*: *CI* Confidence interval, *HIV* Human immunodeficiency virus, *HOMA-B* Homeostatic model assessment of beat cell function and *HOMA-IR* Insulin resistance

^a^Adjusted means and differences are based on log10-transformed variables. Hence, the means presented are therefore geometric means and the differences should be interpreted as ratios

In a sub-group analysis on impact of protein source, we compared the effect of LNS/soy to LNS/whey and found that those who received LNS/whey had a higher HbA1c 2.5 mmol/mol (B 2.5 mmol/L, 95% CI 4.9, 0.2 *p* = 0.036) as compared to soy, but with no difference for FPG, 30-min glucose, 120-min glucose, fasting insulin, HOMA-B, and HOMA-IR (Supplemental table 2).

In a sensitivity analysis among those with good adherence (≥75%), we did not find significant change in the effect of supplementation on glucose parameters except for HbA1c tended to be higher (2.5 mmol/mol, 95% CI 0.1, 4.8, *P* = 0.038) in the supplemented group with good adherence to LNS (data not shown).

## Discussion

There are several studies linking ART and HIV to metabolic syndrome and diabetes, but there are no prior studies assessing the effect of LNS supplementation on glucose metabolism and insulin function among people with HIV. Among adult Ethiopian with HIV starting ART, we found that concomitant LNS supplementation during the first 3 months of ART led to increase in HbA1c, 30-min glucose, 120-min glucose, and fasting insulin compared to ART without supplementation. As a consequence, we also found LNS linked to higher beta-cell function and more insulin resistance. All participants were recruited based on previous ART guidelines and hence generally more immunocompromised compared to current guidelines. Due to study design, we did not include people with HIV with BMI < 17 kg/m^2^.

The participants in the LNS group received an additional 4600 kJ energy-dense supplementation to their regular diet that potentially could lead to increased glucose and insulin resistance [21, 22]. From the total energy in the supplement, 24% was carbohydrate. This could alter insulin action mainly by affecting free fatty acid levels [23]. Meal having high carbohydrate with high glycemic index could increase insulin secretion potentially reduce insulin secretion due to islet amyloid deposition [23]. Moreover, insulin resistance can be induced by niacin intake which is a constituent of the LNS provided in our study [22, 24]. Although the supplementation had potential harmful effect on glucose metabolism, we did not see a higher proportion of diabetes in the supplemented group, thus changes seen in the study period were within the normal range.

HbA1c is a marker for long-term glucose exposure [21] and a now preferred method for diabetes diagnosis as it demands only one test, not dependent on fasting and less affected by acute illness. We found a marginally significant increase in HbA1c in the supplemented group as compared to the non-supplemented group. This change corresponded with the effect from LNS on glucose.

By doing OGTT combined with insulin measures we were able to assess insulin function related to secretion (HOMA-B) and resistance (HOMA-IR), and investigate whether insulin resistance is central/hepatic (based on fasting samples) or systemic/muscular (based on kinetics during OGTT). We found that LNS led to a higher level of fasting insulin. The insulin increase in the supplemented group could be due to an improvement in insulin secretion; however, as we found concomitant increase in HbA1c and glucose during OGTT without change in FPG we speculate that the early, high-energy LNS supplementation primarily leads to peripheral insulin resistance. This said, the higher fasting insulin combined with unchanged FPG is the reason for the increasing insulin resistance by HOMA seen in the LNS group, which is consistent with more hepatic insulin resistance [25] . This said, we can only speculate whether such change will increase long-term risk of diabetes.

Ingestion of proteins, including whey and soy, has been linked to increased insulin secretion among healthy adults, postmenopausal obese women and individuals with type 2 diabetes [26–28]. This effect is most pronounced with whey as it induces glucagon like protein-1 (GLP-1), which further stimulates insulin secretion leading to reduced postprandial hyperglycemia and appetite suppression [26, 29]. Current knowledge on the effect of LNS/soy on glucose during an OGTT has shown heterogeneous results [30]. There are no studies on the effect of combined LNS and whey on glucose metabolism, but a study by Nilsson et al among healthy adults reported that whey protein increased 30-min glucose levels [29]. A meta-analysis suggests that the form and timing of protein intake in relation to meals is crucial to stimulate gut peptides that will in turn lead to glucose lowering and appetite suppressing effect [26]. As insulin secretion is an adenosine triphosphate (ATP) dependent process [31], we speculate that an insulinogenic effect of proteins may be altered among people with HIV initiated on ART as nucleoside reverse transcriptase inhibitors (NRTIs) reduce ATP production potentially causing islet cell mitochondrial toxicity. Even short-term exposure could cause mitochondrial respiratory complex toxicity [32]. We have previously seen that LNS/soy led to relative more fat mass [13], which potentially could cause increasing insulin resistance and hence hyperglycemia as has been reported among non-HIV individuals [24]. Soy ingestion is considered insulinogenic and hence having a glucose lowering effect [33, 34]. This said, we did see an overall effect of the supplementation on insulin markers, but we did not see any specific protein source related effect of neither of the outcomes and we suggest that the major contribution to dysglycaemia from the LNS was from the carbohydrate source, although we are unable to separate the effects.

Apparently, the main increase in HbA1c was seen among those receiving LNS combined with whey as the protein source. Only few non-HIV studies have evaluated the effect of either soy or whey on glucose metabolism, but their results are inconclusive [27, 28]. A randomized cross over trial where whey/guar was provided prior to a carbohydrate rich meal among individuals with type 2 diabetes showed a reduction in glucose and HbA1c levels [35]. As we did not see any effect on protein source on other outcomes than HbA1c, the whey effect needs to be tested in a different population.

Various low-income countries have adopted nutritional support programs in their HIV programs mainly using peanut-based LNS, [36]. If LNS truly increase the risk for dysglycaemia or diabetes, reformulating LNS by reducing carbohydrates should we assessed for safety among vulnerable individuals with HIV. Providing diabetes screening for all individuals initiating LNS should be implemented in clinical practice if at all feasible as it may impact the long-term prognosis for the individual with HIV.

Our study was limited by missing values on HbA1c due to logistical issues. We measured HbA1c using the boronate fluorescence quenching technology which measures not only glycation of N-terminal valine on β-chain, but also β-chains glycated at other sites and glycated α-chains. This method has a high coefficient of variability [25] which could lead to imprecise results and hence a lower power. In this study, adherence to LNS was imperfect (median adherence = 86%), the interpretation of our findings could be affected by adherence to LNS. Use of euglycemic-hyperinsulinemic and hyperglycemic clamps are the golden standard to assess insulin and glucose metabolism and could be used in future studies to provide more accurate estimates of insulin function in HIV. As the supplement in both arms had high carbohydrate content, in addition to whey or soy, we were unable to determine whether dysglycaemia was caused by high carbohydrates or proteins alone or both in the supplemented groups.

## Conclusion

Evaluation of the effects of LNS on metabolic markers among Africans with HIV has not previously been done. We studied the effect of 3 months concomitant LNS and ART intake on glucose and insulin metabolism and found that LNS led to more insulin resistance, higher glucose and HbA1c, which potentially could lead to metabolic disorders. We know people with HIV are at increased risk of NCDs, thus our data are useful as part of a safety evaluation of lipid-based supplementation as it has been included into the national nutrition guidelines of numerous countries including Ethiopia [37]. It is essential to determine whether LNS may lead to insulin resistance and later onset of diabetes and should be explored in a trial designed to test for long-term safety. As we did not assess whether the metabolic changes from LNS supplementation were harmful or beneficial, the long-term effect of intake of lipid-based supplements on glycaemia and other metabolic parameters needs to be evaluated such as diabetes or other NCD onset and related complications. Finally, as our data suggested a whey effect on HbA1c, a potential protein source effect should also be evaluated in other populations.

## Supplementary Information

**Additional file 1: Supplemental table 1.** Baseline characteristics of 268 HIV patients at time of initiation of antiretroviral therapy randomized to lipid-based nutrient supplements with whey or soy as protein-source, or no supplementation. **Supplemental table 2.** Effect of protein-source (whey vs soy) as supplement on change in the glucose and insulin markers among 179 HIV patients after 3 months of concomitant antiretroviral therapy initiation and lipid-based nutrient supplements.

## Data Availability

The Datasets used and/or analyzed for this manuscript are available from the corresponding author on reasonable request.

## Abbreviations

ART: Antiretroviral therapy
ATP: Adenosine triphosphate
BMI: Body mass index
CRP: C-reactive protein
FPG: Fasting plasma-glucose
GLP-1: Glucagon like protein-1
HbA1c: Glycated hemoglobin
HIV: Human immunodeficiency virus
HOMA-B: Homeostatic model assessment index for beta-cell function
HOMA-IR: Homeostatic model assessment index for insulin resistance
LNS/soy: Soy
LNS/whey: Whey
LNS: Lipid-based nutrient supplements
NCDs: Non-communicable diseases
NRTIs: Nucleoside reverse transcriptase inhibitors
OGTT: Oral glucose tolerance test
WHO: World Health Organization
