# Supplementary material for: Effects of nutritional supplementation on glucose metabolism and insulin function among people with HIV initiating ART
Source: BMC Nutr. 2021 Oct 18;7:60. doi: 10.1186/s40795-021-00462-y (PMC8521983; doi:10.1186/s40795-021-00462-y)
Supplement: Supplementary file 1 — Additional file 1: Supplemental table 1. Baseline characteristics of 268 HIV patients at time of initiation of antiretroviral therapy randomized to lipid-based nutrient supplements with whey or soy as protein-source, or no supplementation. Supplemental table 2. Effect of protein-source (whey vs soy) as supplement on change in the glucose and insulin markers among 179 HIV patients after 3 months of concomitant antiretroviral therapy initiation and lipid-based nutrient supplements. [file 40795_2021_462_MOESM1_ESM.docx]

| Supplemental table1. Baseline characteristics of 268 HIV patients at time of initiation of antiretroviral therapy randomized to lipid-based nutrient supplements with whey or soy as protein-source, or no supplementation. | | | |
| --- | --- | --- | --- |
| Characteristics | **Non-supplemented**  **(n = 89)** | **LNS/whey**  **(n = 87)** | **LNS/soy**  **(n = 92)** |
| Age (years) | 32 ±9 | 32 ±8 | 35 ±10 |
| Women, n (%) | 63 (70.8) | 57 (65.5) | 60 (65.2) |
| Educational status |  |  |  |
| No schooling, n (%) | 27 (30.3) | 18 (20.7) | 22 (23.9) |
| Primary education, n (%) | 44 (49.4) | 51 (58.6) | 52 (56.5) |
| Secondary and higher education, n (%) | 18 (20.2) | 18 (20.7) | 18 (19.6) |
| Body mass index (kg/m^2^) | 19.8 ±2.1 | 19.9 ±2.2 | 19.8 ±2.5 |
| Weight (kg) | 51.4 ±7.3 | 54.4 ±7.2 | 54.0 ±7.6 |
| Waist circumference (cm) | 71.9 ±6.7 | 73.4 ±6.0 | 72.7±6.9 |
| C- reactive protein (mg/L)† | 2.1 (0.6-7.5) | 1.9 (0.5-7.0) | 1.3 (0.5-5.3) |
|  |  |  |  |
| HIV-related characteristics |  |  |  |
| WHO stage |  |  |  |
| Stage I, n (%) | 25 (28.4) | 32 (67.8) | 29 (31.5) |
| Stage II, n (%) | 26 (29.6) | 28 (32.1) | 30 (32.6) |
| Stage III, n (%) | 29 (33.0) | 20 (23.0) | 24 (26.1) |
| Stage IV, n (%) | 8 (9.1) | 7 (8.1) | 9 (9.8) |
| HIV viral load (log (1+ copies/mL)) | 4.7 ±0.8 | 4.8 ±0.8 | 4.7 ±0.8 |
| CD4 count (cells/µl) | 191 ±107 | 178 ±90 | 192 ±111 |
| CD8 count (cells/µl) | 878 ±416 | 869 ±405 | 873 ±447 |
| Abbreviation: Human immunodeficiency virus (HIV), Lipid-based nutrient supplements (LNS), World Health Organization (WHO)  *Data are means (±Standard deviation) or number (%)  †Data presented with median with interquartile range | | | |

| Supplemental table 2. Effect of protein-source (whey vs soy) as supplement on change in the glucose and insulin markers among 179 HIV patients after 3 months of concomitant antiretroviral therapy initiation and lipid-based nutrient supplements | | | | |
| --- | --- | --- | --- | --- |
|  | **LNS/whey**  **(reference, n = 87)** | **LNS/soy**  **(n = 92)** | **Difference** |  |
|  | **Adjusted means**  **(95% CI)** | **Adjusted means**  **(95% CI)** | **B**  **(95% CI)** | **P value** |
| Glycated hemoglobin, mmol/mol | 37.4 (35.7; 39.0) | 34.7 (33.3; 36.2) | -2.6 (-0.4; -4.8) | 0.020 |
| Plasma-glucose, mmol/l |  |  |  |  |
| Fasting | 5.8 (5.6; 5.9) | 5.8 (5.7; 6.0) | 0.02 (-0.2; 0.2) | 0.825 |
| 30 minutes | 8.0 (7.7; 8.3) | 7.8 (7.6; 8.1) | -0.2 (-0.6; 0.2) | 0.350 |
| 2 hours | 6.6 (6.3; 6.9) | 6.7 (6.4; 7.1) | 0.2 (-0.3; 0.6) | 0.498 |
|  | **Adjusted log means**  **(95% CI)** | **Adjusted log means**  **(95% CI)** | **10^B^**  **(95% CI)** | **P value** |
| Fasting plasma-insulin, µIU/ml* | 5.5 (4.5; 6.9) | 4.8 (3.9; 5.9) | 0.87 (0.63; 1.16) | 0.315 |
| HOMA-B* | 52.9 (42.3; 66.0) | 43.8 (35.5; 54.1) | 0.83 (0.61; 1.13) | 0.230 |
| HOMA-IR* | 1.4 (1.1; 1.8) | 1.2 (1.0; 1.5) | 0.87 (0.63; 1.19) | 0.371 |
| Abbreviations: Confidence interval (CI), Human immunodeficiency virus (HIV), Lipid-based nutrient supplements (LNS). Homeostatic model assessment of beat cell function (HOMA-B) and insulin resistance (HOMA-IR)  Beta estimates are presented after a linear regression with 95% confidence interval adjusted for age, sex, educational status and baseline status of outcome variables.  *Adjusted means and effect estimates are back-transformed from log 10 scale, the difference should be interpreted as a fraction  Data available: HbA1c: 227 at baseline and 192 at 3 months. Fasting: 264 at baseline and 249 at 3 months. 30m-PG: 264 at baseline and 248 at 3 months. 2h-PG: 265 at baseline and 247 at 3 months. Plasma fasting insulin: 265 at baseline and 246 at 3 months.  HOMA-B: 262 at baseline and 246 at 3 months. HOMA-IR: 263 at baseline and 246 at 3 months | | | | |
